# 2604. Contact With School-Aged Children is a Major Risk Factor for Pneumococcal Colonization in Older Adults

**DOI:** 10.1093/ofid/ofad500.2218

**Published:** 2023-11-27

**Authors:** Anne L Wyllie, Devyn Yolda-Carr, Maikel Hislop, Sidiya Mbodj, Harold J Kennedy, Molly McLaughlin, Loren Wurst, Pari Waghela, Chikondi Peno, Ronika Alexander-Parrish, Adriano Arguedas, Bradford D Gessner, Daniel M Weinberger

**Affiliations:** Yale School of Public Health, New Haven, Connecticut; Yale School of Public Health, New Haven, Connecticut; Yale School of Public Health, New Haven, Connecticut; Yale School of Public Health, New Haven, Connecticut; Yale School of Public Health, New Haven, Connecticut; Yale School of Public Health, New Haven, Connecticut; Yale School of Public Health, New Haven, Connecticut; Stanford Uviversity, San Jose, California; Yale School of Public Health, New Haven, Connecticut; Pfizer, Inc, Bowie, MD; Pfizer, Collegeville, Pennsylvania; Pfizer Biopharma Group, Collegeville, Pennsylvania; Yale School of Public Health, New Haven, Connecticut

## Abstract

**Background:**

Important questions remain about the role of older adults in driving transmission of pneumococcus in the community. This is a critical question for understanding the potential indirect effects of using pneumococcal conjugate vaccines (PCVs) in older adults. For non-institutionalized individuals, the most likely source of adult-to-adult transmission is in the household. The goal of this study was to characterize the dynamics and risk factors for acquisition of pneumococcus in older adults.

**Methods:**

We designed a longitudinal study to sample adults > 60 years of age living in the same household (New Haven, CT), and without younger contacts residing in the household. Saliva samples and questionnaires regarding social behaviors and health status were obtained every 2 weeks for a period of 10 weeks. DNA extracted from culture-enriched saliva was tested using qPCR for pneumococcus genes *piaB* and *lytA*.

**Results:**

Across the two study seasons (November 2020-August 2021, November 2021-September 2022), 184 individuals from 93 households were sampled and completed all 6 visits. Overall, 31/184 (16.8%) individuals were colonized on at least one time point based on qPCR detection of *piaB*. Two individuals were colonized at 6/6 time points, and two others were colonized at 5/6 time points during the 10-week sampling period. In 7/93 (7.5%) households, both members were colonized, though not necessarily at the same time point. Pneumococcal colonization was substantially higher among individuals who had contact with children < 18 years as compared to those with no recent contact with children (9.4% vs 2.3%). Participants who reported recent contact with < 5 year olds and 5-9 year olds had particularly elevated prevalence (14.3%; 13.0%, respectively).

**Conclusion:**

Pneumococcal acquisition related to adult-to-adult transmission is low. Contact with pre-school and young school-aged children was the most important factor that influenced acquisition rates of pneumococcus among older adult households.

**Disclosures:**

**Anne L. Wyllie, PhD**, Co-Diagnostics: Board Member|Merck: Grant/Research Support|Pfizer: Advisor/Consultant|Pfizer: Grant/Research Support **Ronika Alexander-Parrish, RN, MAEd**, Pfizer, Inc.: Employee|Pfizer, Inc.: Stocks/Bonds **Adriano Arguedas, Medical director**, Pfizer: Emplyee|Pfizer: Stocks/Bonds **Bradford D. Gessner, M.D., M.P.H.**, Pfizer: I am an employee of Pfizer|Pfizer: Stocks/Bonds **Daniel M. Weinberger, PhD**, GSK: Advisor/Consultant|Merck: Advisor/Consultant|Merck: Grant/Research Support|Pfizer: Advisor/Consultant|Pfizer: Grant/Research Support

